# A Comparative View of the Modern Medical Theories, &c.

**Published:** 1799-07

**Authors:** N. P. Gilbert


### *A Comparative View of the Modern Medical Theories, &c.* *By* N. P. Gilbert.

[*Concluded from our laft Number,* pp. 360—363.]

In Germany, Haen and Stoll fhared the public opinion; both were inceffantly employed in fimplifying the materia medica, and both contributed to the banifhment of that prodigious number of medicinal fubftances which had obtained fo much credit in that country; but the authority of Boerhaave was ftill the only one that could be relied on with fafety. It has been faid, and with fome truth, that one of the traits which chiefly diftinguifhed that great man, was his having infpired thofe who adopted his doctrines with an enthufiaftic veneration for his opinions.

In Great Britain, the genius of Cullen long fince prepared a revolution in medicine. At the inftant his works appeared they attracted every mind by the eloquence, the method, and the force of reafoning of the author.

For

For definitions always imperfect, he substituted descriptions, pictures which HIPPOCRATES himself would not have disowned, a nosological method, a model of precision and perspicuity, which deserved to be placed above that of the celebrated Sauvages, of which it is nevertheless only the counterpart; but Cullen, that excellent practitioner, that accurate nosologist, could not guard himself from the mania of explaining the proximate causes of diseases. From this time he abandoned the path of observation, and wandered in his turn into the boundless field of ætiology. After having overturned the physical and chemical laws of Boerhaave, and the psychological laws of Stoll, he declared in favour of HOFFMAN; he placed his theory in a new light, or rather improved upon his ideas without introducing them into his work. In the mean time, his reputation was not confined to England, his works crossed the seas, and were received with enthusiasm in the most celebrated universities of Europe. In France, at this period, a medical system prevailed, without doubt the most perfect, as it admitted in the animal economy a principal preserver and restorer of the functions, to which all the spontaneous movements appear to be attached in the states of health and disease. This sublime idea of the *vis medicatrix* of nature, acknowledged by HIPPOCRATES, but which the sectaries had either overlooked or despised, and which was represented under distorted forms by the impetuous VAN HELMONT, afterwards reproduced by Stoll, under the idea of the intelligent principle, and revived by Cullen, under the title of reaction of the system, and at length reduced to its true state by the interesting labours of the French physicians, owed its reputation and glory to the powerful influence of philosophy, and the analysis of the sciences, literature, and the arts. Indeed, this doctrine was acquired by assiduous labour; in referring to the system of Hippocrates, it imposed the law never to swerve from the path adopted by that great man; but at the same time it fatigued the indolent and disgusted the superficial.

These difficulties procured the theory of Boerhaave a great number of proselytes. Such was the state of medicine in France when the revolution, that immense movement of persons and things, suddenly occupied the minds of men. At that time, a new medical theory was framed in France, which, about seven or eight years since, was introduced in England, where it obtained great credit: it was from thence transferred to Germany, where it apparently acquired the most brilliant reputation, and was finally received in Italy with an almost general enthusiasm. I allude to the Brunonian theory. It would be difficult to say upon what foundation it has been possible to present it as a new doctrine; for, in establishing excitability as a peculiar faculty for the exercise of the functions of the animal economy, BROWN has only united in his system, irritability and sensibily,—properties of the human being so admirably delineated by the illuſtrious HALLER, and the ineſtimable DE CEZE.

What have rendered this doctrine intereſting, are the invaluable advantages which it holds out to man in a ſuffering ſtate, in delivering him from every tedious and painful method of cure, in regulating, at all times, his ſenſitive powers and inclinations. This doctrine is likewiſe ſeducing, by the extreme ſimplicity which it pretends to have introduced into the curative art; ſince by it the whole of medical ſcience is reduced to the following ſimple propoſition:—Excitability is the property which characteriſes life. Excitement, or the action of the living powers, conſtitutes perfect health by conſuming only a certain due portion of excitability. Too violent an excitement, by waſting too readily the excitability, generates inflammatory diſorders and requires debilitating medicines. Too weak an excitement, by accumulating excitability, produces diſeaſes of debility, and renders the uſe of exciting medicines neceſſary. In whatever ſtate of the body, whether healthy or diſeaſed, there always exiſts either too ſtrong or too weak an excitment. Hence there can be only two ſpecies of diſeaſes, two methods of treatment, two kinds of medicinal ſubſtances, ſuch, in a few words, is the baſis of the theory of Brown. But can we admit a doctrine which conſiders life as a forced ſtate, which does not acknowledge the automatical action of nature in diſeaſes? Can we admit the truth of a doctrine, which reſts entirely on one ſingle property of the living ſubject, and which depreciates all the dogmas it profeſſes reſpecting the hypothetical eſtabliſhment of a ſingle principle?

It is too true, that the theory of Brown could not ſuſtain the ſhock of a profound diſcuſſion, and that his moſt violent adverſaries have ſtarted objections to him, which ſtill remain unanſwered. Nevertheleſs, if it lead to practical obſervations, if it approach to the true method of making medical obſervations, if it admit of being compared with theſe, the doctrine of Brown is only to be looked upon as a collection of propoſitions, applicable to practical medicine. How many important truths has it not reſtored to mankind, which would otherwiſe have been conſigned to oblivion? What a ſubject for meditation is offered to clinical medicine, by this elegant diſtinction of weakneſs into abſolute debility, feebleneſs of infancy, cauſed by a too faint ſtimulation of the accumulation of excitability; and by indirect weakneſs, or the exhauſtion of the power of excitement, the melancholy and too common conſequence of debauchery, and the abuſe of the ſenſual paſſions? What lights will not clinical medicine receive from this diviſion of diſeaſes, from ſpecific actions in which the whole affected ſyſtem calls for general remedies, and in local internal maladies; of various ſyſtems which only preſent ſpecial indications to be anſwered. If the dangers of repeated purgatives in intermittent fevers, if the neceffity of a ftimalating medicine, in moft chronic difeafes, be at this time inconteftable points of doctrine, it is perhaps to the theory of Brown, that we are indebted for thefe fortunate changes in the practice of phyfic. Hence it is true, that this theory, which by the vague generalities which it propofes for practice, by the introduction of whimfical ideas, by the obfcure, and often enigmatical language, which it gives rife to; did not appear worthy a place among the fyftems of medicine, which perhaps will again be confulted with pleafure, with intereft, and with utility by philofophic phyficians; and may yet afford them great affiftance in the curative art.

The application of modern chemiftry, to pathology and therapeutics, or rather that part of the fyftem of the fcience of man, which is connected with chemiftry, in the different ftates of health and ficknefs, is what remains for me to examine. The author of this new theory, is a much-refpected phyfician, whofe labours have confiderably enriched medical literature, and thrown much light on the practice of phyfic. The following are a few of its principles.

The various actions of life prefent, in organized bodies, an uninterrupted fucceffion of fluids throughout them of fluids in folid bodies; now thefe changes cannot operate on the fubftances, which enter into the compofition of the living fubject, without altering the conftituent ftate of the elementary materials of all thofe fubftances. Hence it appears, that oxygen, hydrogen, carbon, phofphorous, fulphur, azote, and lime, are thrown out every inftant by health and difeafe, and form new combinations, which may tend to determine the principal caufe or nature of pathological affections. Thus, the inflammatory difeafes of Stoll, or, which is the fame thing, the fthenic diforders of Brown, are, in the theory of Girtanner, of Rolla, and of Baumes, only a perfect fuperoxygenation of the living fyftem, a quick combuftion, accompanied by its fenfible phenomena, the difcharge of caloric, the production of a very intenfe animal heat, and an increafed energy of the vafcular arterial fyftem. The author fupports his doctrine on feveral pofitive experiments made by Bertholet, Fourcroy, Chaptal, Parmentier, Deyeux, and Vauquelin, to whofe labours true chemiftry is under fuch great obligations. Hence fcorbutic diforders, which, according to the doctrine of Stoll, only introduced the feptic change of the animal fluids, but according to Brown, which are only the produce of the laft ftage of direct afthenia, are, in the language of modern chemiftry, a greater or lefs difoxygenation of the albuminous juices.

If the portion of oxygen neceffary to the maintenance of life, decreafe; if this principle of irritability is weakened, being fubject to new combinations in the elementary materials of the living body, it caufes a fuperabundance of hydrogen and carbon.

The

The elements of bile then predominate in the blood; bilious fevers are the confequence, always accompanied by that kind of acrid heat, which difcloses the prefence of carbonated hydrogen. Such is the new theory of putrid difeafes.

In feveral other pathological ftates, phofphorization has a powerful influence. This agent is known to be the generative caufe of feveral difeafes of the bones, as rheumatic and arthritic affections, the ætiology of which has hitherto remained undifcovered. The therapeutic art has alfo been fubmitted to this chemical theory; we need only acknowledge in medical fubftances the fuperoxygenates, the difoxygenates, the hydrogenates, the azotes, the phofphorates, &c. &c. &c.

However ingenious this theory may feem, it has this great defect, that any direct comparative experiment made on the living fubject in a ftate of health or difeafe, will not conduce to its eftablifhment; befides, it reproduces, in an indeterminate degree, the fyftem of humoral pathology, of which philofophic phyficians had wifely circumfcribed the bounds; the explanation of phenomena does not devolve on any fact inconteftibly eftablifhed; but that which will probably prevent the admiffion of it by phyficians, is, that it deprives the animal economy of its greateft and moft diftinctive character, VITALITY, that principal preferver and renovator of beings, that wonderful quality which fuftains them againft phyfical power which continually tends to their decompofition, which acts independent of mechanical, phyfical, or chemical laws, and which leaves no other traces of its prefence in the body than what is expofed by the motion of life. It is true then, that an ætiology, that a chemical nofology, with difficulty explain the ftate of the animal economy, in the various affections which can produce any change in the foundnefs of its functions; yet, if we draw this medical theory from obfervations; if we are contented with regarding it as a combination of experiments, of analogies, and of analyfes, from which the curative art might reap advantage, how many ingenious effays, brilliant difcoveries, happy refources, views really ufeful, falutary medicinal preparations, advantageous modes of healing, have not the labours, the genius, and the application of fo many celebrated chemifts produced in the courfe of a few years? of the birth of whom, France, England, Germany, and Italy claim the honour.

The analytical datails into which I have juft entered relative to the actual medical theories, clearly prove,

That every medical theory which is founded on an hypothefis, ought to be banifhed from amongft the number of ufeful difcoveries in medicine.

That

That the greateſt misfortune which can happen to a patient, is to fall into the hands of a phyſician bigotted to a particular ſyſtem, however ingenious he may be, or whatever conformity there may apparently be between the facts and the explanation which he gives of them.

That it is not medical theory which can furniſh to the philoſophic phyſician, any ſubject for uſeful obſervations.

That the character of a real phyſician is, not to attach himſelf excluſively to any particular theory, but to inveſtigate all, and to collect ſuch advantages as they may offer, by comparing them with medicine of obſervation; that it is this which may be called medical eclectiſm.

This great art of extracting, from all the modern ſyſtems, a medical doctrine, which might contain nothing but what any one of them may declare to be uſeful, would at the preſent time demand the re-union of the greateſt talents. The character of a conſummate phyſician, an indefatigable chemiſt, a philoſophic obſerver, an exact anatomiſt, a practitioner of great experience, an erudite author, an eloquent writer, a profound thinker, all ought to be concentered in him. Such was Boerhaave, when he laid the foundation of his Medical Inſtitutes, and immortal Aphoriſms; a work in which ability is united to method, and the art of writing added to conceptions of the moſt extenſive genius. If the diſcoveries of ſubſequent times have brought back every mind to the doctrine of Hippocrates, to medicine of obſervation, Boerhaave has not the leſs preſerved all his rights to the veneration and eſteem, even of thoſe who have ſubſtituted in lieu of his theory, a philoſophic doctrine more worthy of the preſent age.

Hence it becomes the duty of every medical ſtudent, to reconcile by his eſſays and by his exertions, according to his wiſhes, the time when ſimplified medicine may be reduced to an elementary, conciſe, and rational method, proper to direct inſtruction and enlighten practice. It is the duty of medical ſocieties, eager to concur in this great undertaking, to encourage every work, however imperfect it may be; becauſe, they tend to re-animate emulation, to excite zeal, to light the torch of genius, and thus become the auſpicious commencement of a complete production. Such are the motives which have induced me to trace this faint ſketch, in which I have endeavoured to do juſtice to the Medical Society.

